# Clinical Features of Coexisting Anti-NMDAR and MOG Antibody-Associated Encephalitis: A Systematic Review and Meta-Analysis

**DOI:** 10.3389/fneur.2021.711376

**Published:** 2021-08-26

**Authors:** Jiayue Ding, Xiangyu Li, Zhiyan Tian

**Affiliations:** ^1^Department of Neurology, Tianjin Medical University General Hospital, Tianjin, China; ^2^Department of Neurology, Tianjin Huanhu Hospital, Tianjin, China

**Keywords:** N-methyl-D-aspartate receptor, myelin oligodendrocyte glycoprotein, encephalitis, antibody, meta-analysis

## Abstract

Coexisting anti-NMDAR and MOG antibody (anti-NMDAR-IgG^+^/MOG-IgG^+^)-associated encephalitis have garnered great attention. This study aimed to perform a secondary analysis to determine the clinical features of this disease. We searched several databases for related publications published prior to April 2021. A pooled analysis was conducted with the fixed-effects model using the Mante-Haenszel method (*I*^2^ ≤ 50%), or the random-effects model computed by the DerSimonian–Laird method (*I*^2^ > 50%). Stata software (version 15.0 SE) was used for the analyses. Nine observational studies and 16 case reports (58 cases with anti-NMDAR-IgG^+^/MOG-IgG^+^, 21.0 [8.5, 29.0] years, male 58.6%) were included. The incidences (95%CI) of anti-NMDAR-IgG^+^/MOG-IgG^+^ in the patients with serum MOG-IgG^+^ and CSF anti-NMDAR-IgG^+^ were 0.09 (0.02–0.19) and 0.07 (0.01–0.19), respectively. The median [IQR] of CSF anti-NMDAR antibody titer was 32 [10, 100], and the serum anti-MOG antibody titer was 100 [32, 320]. The prominent clinical symptoms were encephalitic manifestations, including seizures (56.9%) and abnormal behavior (51.7%), rather than demyelinating manifestations, such as speech disorder (34.5%) and optic neuritis (27.6%). Relapse occurred in 63.4% of anti-NMDAR-IgG^+^/MOG-IgG^+^ patients, in whom 50.0% of cases relapsed with encephalitic manifestations, and 53.8% relapsed with demyelinating manifestations. The common MRI changes were in the cortex or subcortex (70.7%) and brainstem (31.0%). 31.3% of patients presented with unilateral cerebral cortical encephalitis with epilepsy and 12.5% displayed bilateral frontal cerebral cortex encephalitis. Anti-NMDAR-IgG^+^/MOG-IgG^+^ patients showed more frequent mental behavior (OR, 95%CI, 68.38, 1.36–3,434.37), involuntary movement (57.86, 2.53–1,325.11), sleep disorders (195.00, 7.07–5,380.15), and leptomeninge lesions (7.32, 1.81–29.58), and less frequent optic neuritis (0.27, 0.09–0.83) compared to anti-NMDAR-IgG^−^/MOG-IgG^+^ patients and presented more common relapse (5.63, 1.75–18.09), preceding infection (2.69, 1.03–7.02), subcortical lesions (116.60, 4.89–2,782.09), basal ganglia lesions (68.14, 2.99–1,554.27), brainstem lesions (24.09, 1.01–574.81), and spinal cord lesions (24.09, 1.01–574.81) compared to anti-NMDAR-IgG^+^/MOG-IgG^−^. In conclusion, anti-NMDAR-IgG^+^/MOG-IgG^+^ was rarely observed, but the incidence rate of relapse was very high. The overall symptoms seemed to be similar to those of NMDAR encephalitis.

## Introduction

Anti-N-methyl-D-aspartate receptor (anti-NMDAR) encephalitis is an immune-mediated disease characterized by the presence of specific cerebrospinal fluid (CSF) IgG antibodies against GluN1 subunits (1). Anti-NMDAR encephalitis (NMDARE) commonly causes a variety of neuropsychiatric symptoms, including psychiatric symptoms, behavioral abnormalities, seizures, speech disorders, and decreased consciousness (2, 3). Although the incidence of the disease is estimated to be 1.5 per million population per year, this entity has garnered great attention in recent years owing to its specific clinical features and laboratory test results that cannot be explained by other, traditional encephalitis disorders (4). With the deeper recognition of NMDARE, some researchers have found that concomitant antibodies play a role in the progression of the disease (5). Myelin oligodendrocyte glycoprotein (MOG) antibody is an important concomitant antibody that may target oligodendrocytes in NMDAREs (6, 7). Moreover, anti-NMDAR antibodies could also be found in MOG antibody disease (MOG-AD) (8).

MOG antibody is initially delineated in demyelinating diseases such as multiple sclerosis, neuromyelitis optica, and acute disseminated encephalomyelitis, and is regarded as an assistant antibody that may worsen outcomes (6, 9). However, the highly sensitive and specific method for MOG antibody detection using cell-based assays (CBA), along with the new diagnostic criteria for similar neuroinflammatory diseases, has made it possible to identify MOG-AD as an independent entity distinct from other demyelinating diseases (10, 11). Therefore, both anti-NMDAR and anti-MOG antibodies are responsible for encephalitis. Additional studies have pointed out that NMDAR and MOG autoantigens may be simultaneously present on the surface of oligodendrocytes, which means that these two types of antibodies can coexist in one patient (12, 13). Some observational studies and case reports have described the characteristics of patients with dual-positive anti-NMDAR and MOG antibodies, and compared them with those of patients with sole-positive anti-NMDAR or MOG antibodies; however, these sample sizes are too small to reach convincing conclusions (5, 7, 8, 14–36). Therefore, this study aimed to summarize the existing publications and perform a secondary analysis to further unravel the clinical features of patients with coexisting anti-NMDAR and MOG antibody-associated encephalitis.

## Materials and Methods

### Search Strategies

The keywords “MOG” or “myelin oligodendrocyte glycoprotein” and “NMDA” or “N-methyl-D-aspartate” were searched in several literature databases, including Medline, Embase, and Cochrane, for publications that were published prior to April 2021. We also reviewed the references of the retrieved articles for additional reports to avoid missing out in our search.

### Study Selection

Observational studies and case reports were involved in this study. The enrolled observational studies were screened using the following criteria: (1) a cohort of patients with coexisting positive anti-NMDAR-IgG and MOG-IgG (anti-NMDAR-IgG^+^/MOG-IgG^+^); (2) and/or a cohort of patients with positive anti-NMDAR-IgG (anti-NMDAR-IgG^+^/MOG-IgG^−^) or MOG-IgG (anti-NMDAR-IgG^−^/MOG-IgG^+^) alone; (3) antibodies were detected using CBA. The enrolled case reports had to comply with the following criteria: (1) patients with anti-NMDAR-IgG^+^/MOG-IgG^+^; (2) antibodies were detected using CBA. Anti-NMDAR-IgG^+^ indicated CSF reactivity against NMDAR, while MOG-IgG^+^ indicated serum reactivity against MOG, which followed the NMDARE and MOG-AD diagnostic criteria. Patients with serum anti-NMDAR-IgG^+^ or CSF MOG-IgG^+^ were excluded from the final screening.

### Data Extraction

The following information was retrieved for each article: study design, demographics, clinical features, MRI images, and outcomes. Data was collected by three reviewers (JY-Ding, XY-Li, and ZY-Tian), and if inconsistency occurred between the two reviewers, a third reviewer would re-examine the data, and the final decision was made based upon the majority, with at least two thirds of the votes.

### Statistical Analysis

Stata software (version 15.0 SE) was used for analysis in this study. The values are presented as mean ± standard deviation or median [interquartile range, IQR] for continuous data, and as number (percentage, %) for categorical data. Pooled analysis was conducted with fixed-effects model using Mantel–Haenszel method when the heterogeneity was expected to be available (*I*^2^ ≤ 50%). Otherwise, the random-effects model computed through the DerSimonian-Laird method was performed (*I*^2^ > 50%). The pooled results were shown as odds ratio (OR) with 95%CI for the categorical data. *P* < 0.05 were considered statistically significant. The pooled incidence rate along with 95%CI were calculated using double inverse sine transformation.

## Results

### Search Results

**Figure 1:**
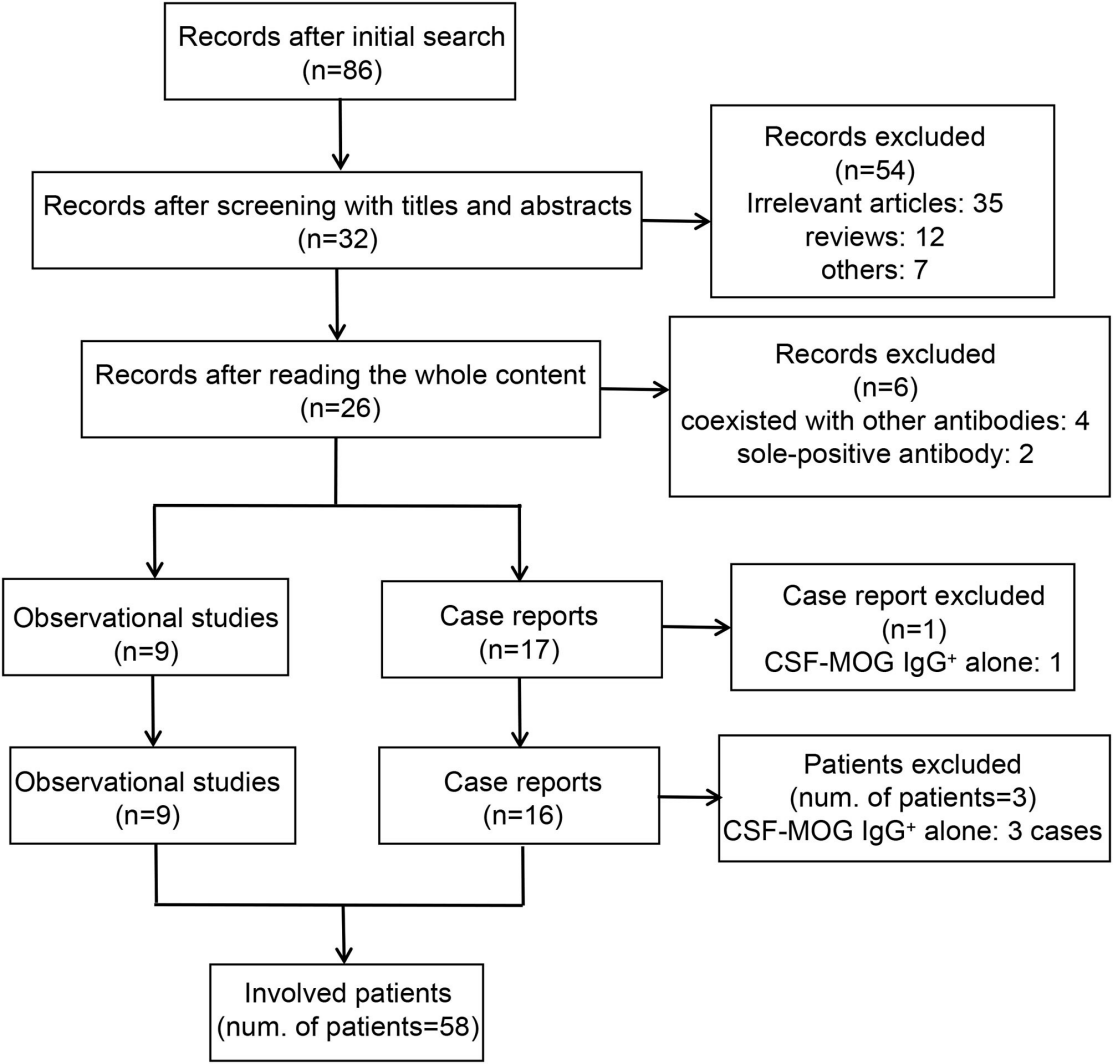
Study selection flowchart.

The adopted search strategy identified 86 articles published before April 2021 in the initial search. During the screening process, 54 articles were excluded after screening the titles and abstracts, and six articles were removed after thoroughly reading the whole content. None of the articles were involved after reviewing the references of the retrieved articles. One case report (Fujimori et al.) that reported a case of CSF MOG-IgG^+^ alone was excluded (14). A total of 25 articles, including nine observational studies and 16 case reports, were included in this systematic analysis (5, 7, 8, 15–36). Fifty-eight out of 61 patients were enrolled in this review, as the remaining three patients were CSF MOG-IgG^+^ alone (Ren et al.: one patient; Du et al.: two patients) (25, 34). A flowchart of the screening process is presented in Figure 1.

### The Incidence of Anti-NMDAR-IgG^+^/MOG-IgG^+^

**Figure 2:**
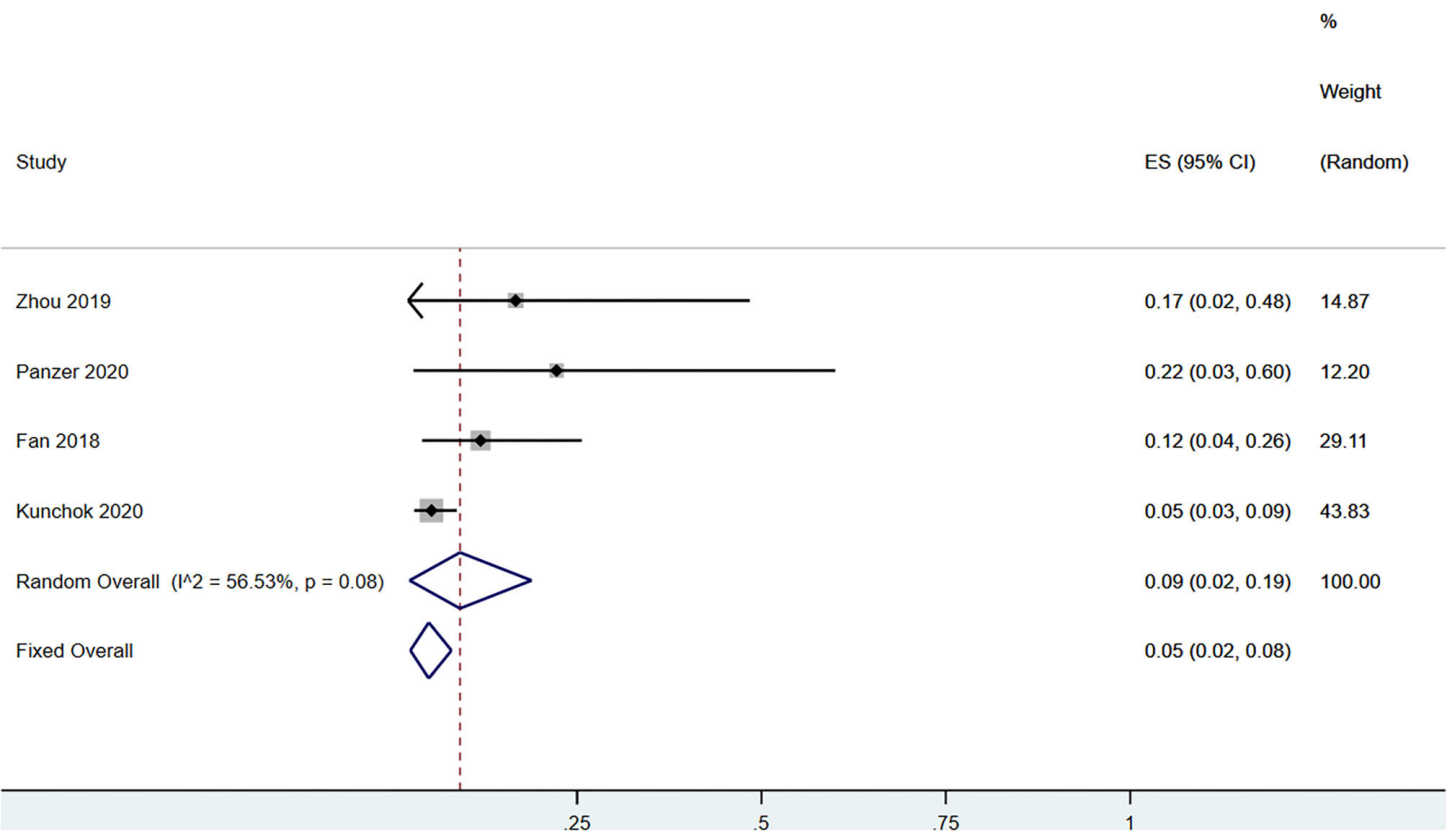
The pooled incidence rate of CSF anti-NMDAR-IgG^+^ in the patients with serum MOG-IgG^+^.

**Figure 3:**
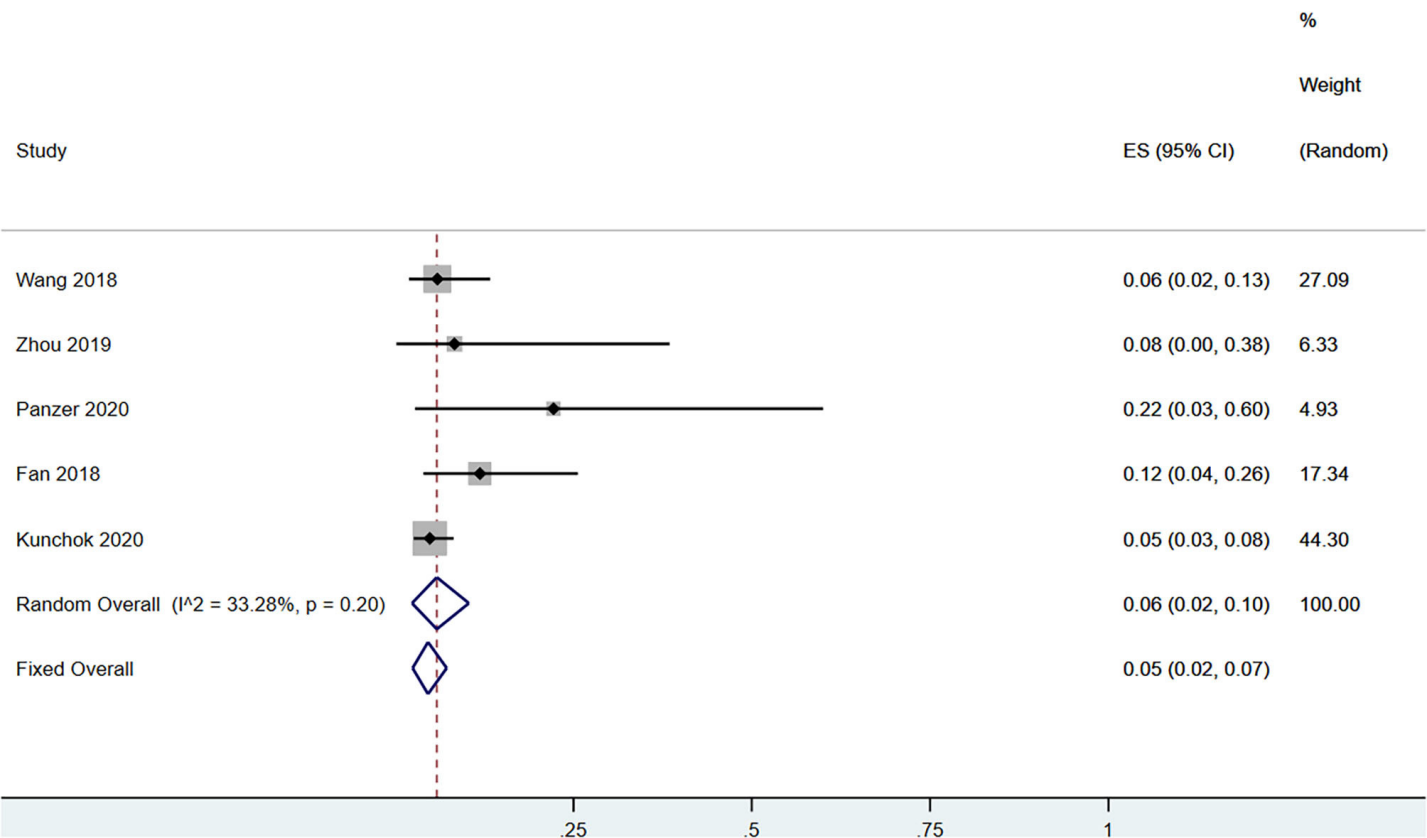
The pooled incidence rate of NMDARE in the patients with serum MOG-IgG^+^.

**Figure 4:**
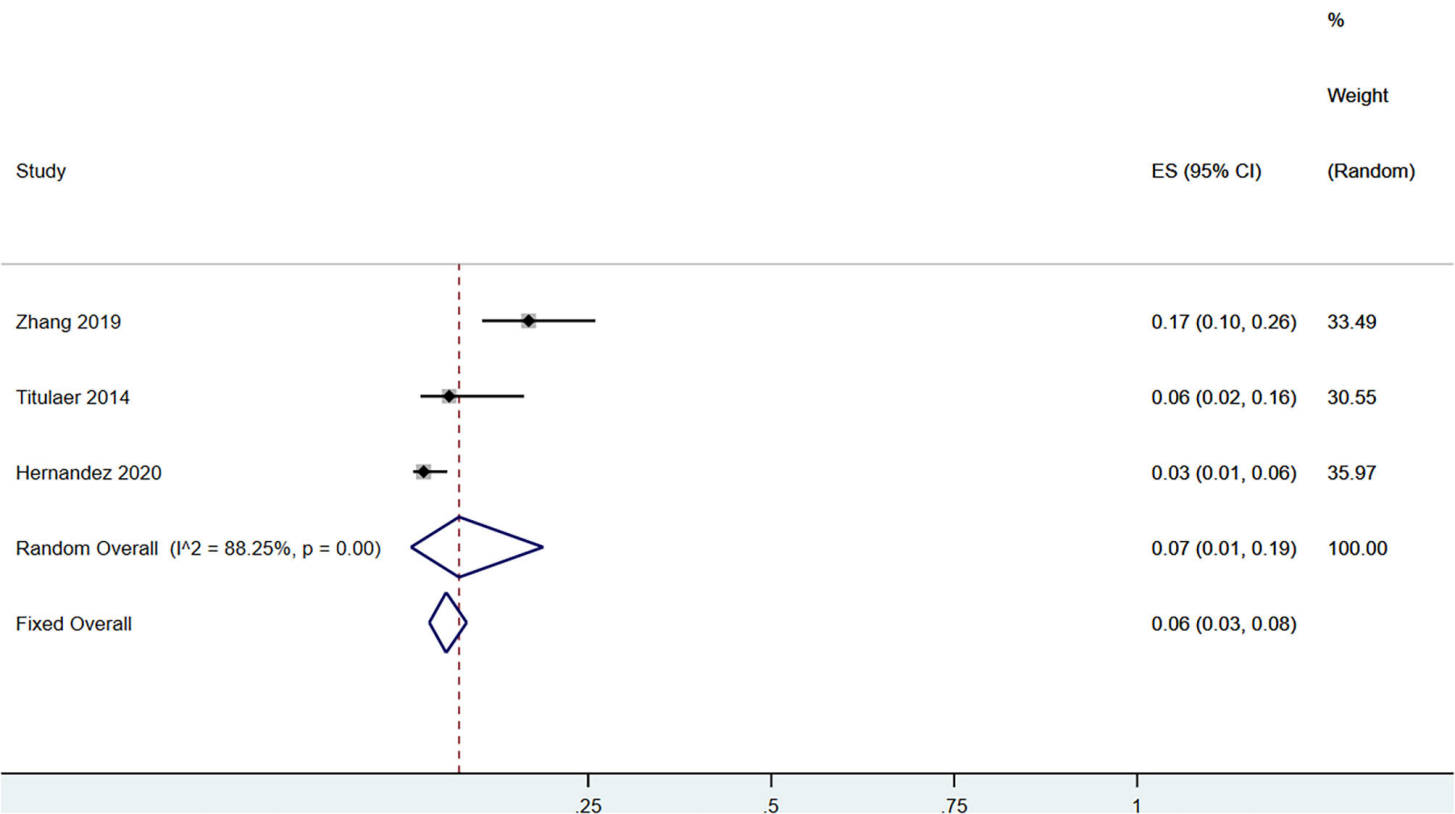
The pooled incidence rate of serum MOG-IgG^+^ in the patients with NMDARE.

A total of 58 patients had CSF anti-NMDAR-IgG^+^ and serum MOG-IgG^+^ simultaneously. Among the patients with serum MOG-IgG^+^ (*n* = 329), 23 patients coexisted with CSF anti-NMDAR-IgG^+^, and the pooled incidence (95%CI) was of 0.09 (0.02–0.19) (enrolling four observational studies, *I*^2^ = 56.53%, thus random pooled model was used, shown in Figure 2). In addition, among the patients with serum MOG-IgG^+^ (*n* = 413), 26 were diagnosed as NMDARE simultaneously, and the pooled incidence (95%CI) was of 0.05 (0.02–0.07) (enrolling five observational studies, *I*^2^ = 33.28%, thus fixed pooled model was used, shown in Figure 3). Among the NMDARE patients (*n* = 369), a total of 27 cases had serum MOG-IgG^+^, and the pooled incidence (95%CI) was of 0.07 (0.01–0.19) (enrolling three observational studies, *I*^2^ = 88.25%; thus, the random pooled model was used, shown in Figure 4).

### Summary of Current Publications on Anti-NMDAR-IgG^+^/MOG-IgG^+^

A total of nine observational studies and 16 case reports have investigated the clinical characteristics of anti-NMDAR-IgG^+^/MOG-IgG^+^. When pooling these studies, 19 patients (median [IQR], 26.5 [17.25, 32.25] years, male 63.2%) were depicted in the case reports and 58 cases (21.0 [8.5, 29.0] years, male 58.6%) in the observational studies plus the case reports. The median [IQR] of CSF anti-NMDAR antibody titer was 32 [10, 100], and the serum anti-MOG antibody titer was 100 [32, 320]. Based on the case reports, five out of 13 patients (38.5%) who were diagnosed with both anti-NMDAR and anti-MOG antibodies at the first attack episode, were found to be dual positive. Other antibodies, such as AMPAR1/2, CASPR2, and GABA_B_R, were negative in both serum and CSF in these patients, except for one patient with comorbid CASPR2 (27).

The clinical symptoms of anti-NMDAR-IgG^+^/MOG-IgG^+^ were as follows: encephalitic manifestations, including seizures (52.6 and 56.9% in the case reports and the observational studies plus case reports, respectively), abnormal behavior (31.6 and 51.7%), headache (63.2 and 29.3%), fever (52.6 and 29.3%), consciousness disorder (36.8 and 25.9%), and sleep disorder (10.5 and 19.0%); demyelinating manifestations, including speech disorder (31.6 and 34.5%), optic neuritis (52.6 and 27.6%), limb weakness (31.6 and 27.6%), paresthesia (31.6 and 22.4%), ataxia (42.1 and 20.7%), cranial nerve destruction (0.0 and 12.1%), dysphagia (15.8 and 6.9%), orofacial dyskinesias (0.0 and 6.9%), and dysarthria (5.3 and 3.4%).

**Figure 5:**
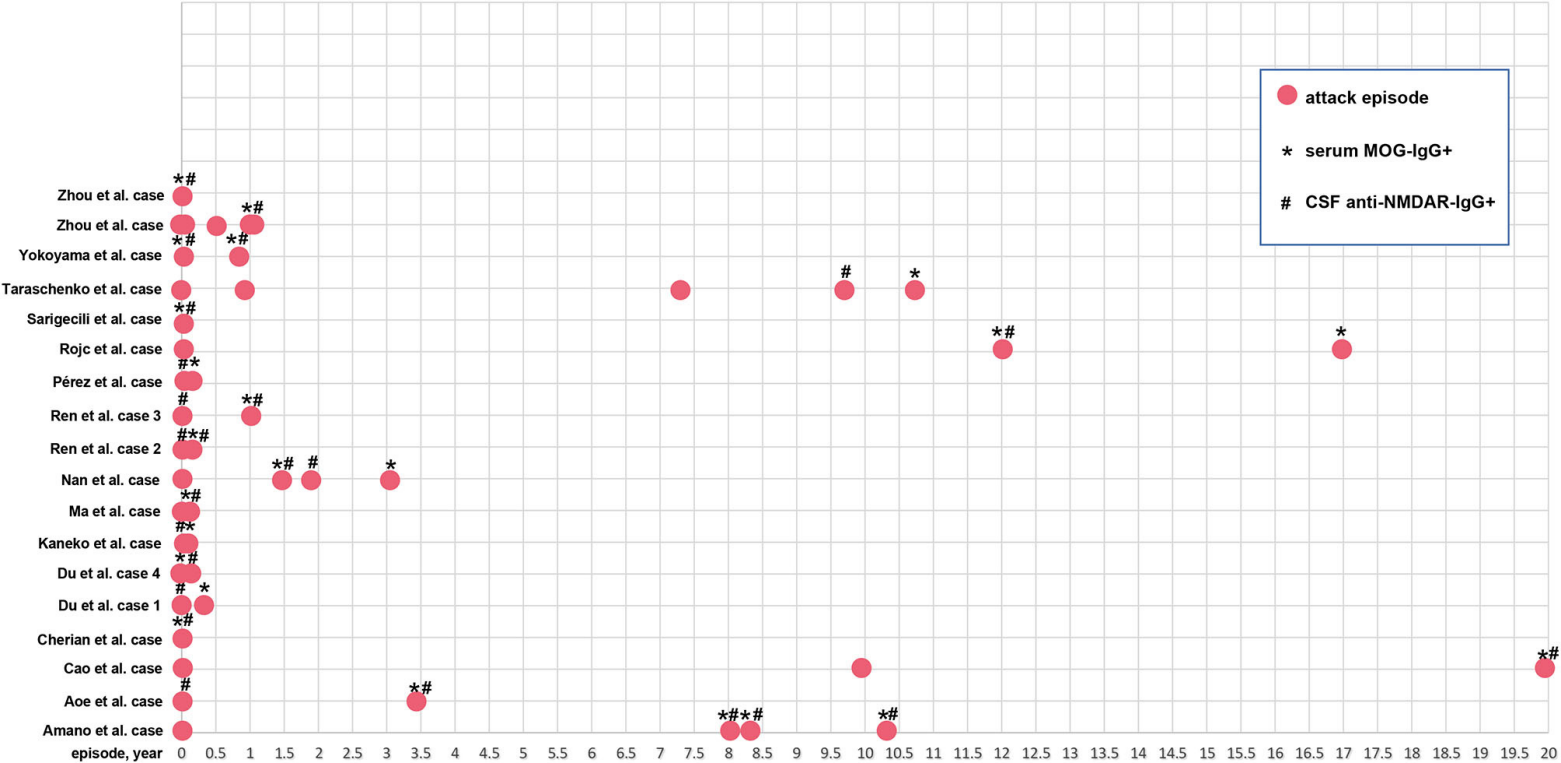
The relapse time point of the 19 patients. Red solid circle indicates an attack or relapse. *Serum MOG-IgG^+^ is detected at this time point; ^#^CSF anti-NMDAR-IgG^+^ is detected at this time point.

Forty-five of 58 patients (77.6%) presented with encephalitic manifestations during the first episode, of whom 23 (51.1%) displayed seizures. Sixteen out of 58 patients (27.6%) presented with demyelinating manifestations at the first attack, and 11 (68.8%) had optic neuritis. A total of 41 patients underwent a follow-up investigation of the relapsed symptoms. Twenty-six patients relapsed (63.4%), of whom 13 relapsed with encephalitic manifestations (four seizures), and 14 relapsed with demyelinating manifestations (10 optic neuritis). Nine out of 26 patients had encephalitic manifestations at the first attack and presented additional demyelinating manifestations at the second episode. Two patients had encephalitic manifestations at the first attack but presented additional demyelinating manifestations at the second episode. In the case reports, 16/19 patients (84.2%) experienced relapse. The longest relapse time was almost 20 years, and 6 cases (31.6%) had more than two episodes. The attack time points for each case are shown in Figure 5.

The common MRI changes were cortex (53.4 and 42.1% in the case reports and the observational studies plus case reports, respectively), brainstem (21.1 and 31.0%), basal ganglia (15.8 and 29.3%), thalamus (10.5 and 24.1%), spinal cord (15.8 and 22.4%), cerebellum (21.1 and 17.2%), optic nerve (15.8 and 17.2%), leotomenings (15.8 and 15.5%), periventricular area (21.1 and 13.8%), corpus callosum (15.8 and 10.3%) and brachium pontis (5.3 and 8.6%). Thirty-two patients had comprehensive imaging information, of whom 10 (31.3%) patients presented with unilateral cerebral cortical encephalitis with epilepsy (UCCEE) and 4 (12.5%) displayed bilateral frontal cerebral cortex encephalitis (BFCCE).

The treatment strategies included as the first-line therapy: intravenous methylprednisolone pulse (IVMP, 100 and 48.3%), intravenous immunoglobulins (IVIG, 52.6 and 56.9%), plasma exchange (PLEX, 10.5% and 8.6%); second-line therapy, rituximab (RTX, 15.8 and 25.9%), cyclophosphamide (CTX, 0.0 and 1.7%), azathioprine (AZA, 10.5 and 5.2%). All of these are shown in Table 1.

**Table 1:** Clinical features of the patients with anti-NMDAR-IgG^+^/MOG-IgG^+^.

**Items (%)**	**Case reports**	**Observational studies plus case reports**
Num. of patients	19	58
Demographics		
Median [IQR] age, year	26.5 [17.25, 32.25]	21.0 [8.5, 29.0]
Male	12 (63.2)	34 (58.6)
Relapse	16 (84.2)	46 (79.3)
Clinical manifestations		
Encephalitic manifestations		
Seizures	10 (52.6)	33 (56.9)
Abnormal behavior	6 (31.6)	30 (51.7)
Headache	12 (63.2)	17 (29.3)
Fever	10 (52.6)	17 (29.3)
Consciousness disorder	7 (36.8)	15 (25.9)
Sleep disorder	2 (10.5)	11 (19.0)
Demyelinating manifestations		
Speech disorder	6 (31.6)	20 (34.5)
Optic neuritis	10 (52.6)	16 (27.6)
Limb weakness	6 (31.6)	16 (27.6)
Paresthesia	6 (31.6)	13 (22.4)
Ataxia	8 (42.1)	12 (20.7)
Cranial nerve destroy	0 (0.0)	7 (12.1)
Dysphagia	3 (15.8)	4 (6.9)
Orofacial dyskinesias	0 (0.0)	4 (6.9)
Dysarthria	1 (5.3)	2 (3.4)
MRI images		
Cortex^*^	8 (42.1)	31 (53.4)
Unilateral	2 (25.0%)	16 (51.6%)
Bilateral	6 (75.0%)	12 (38.7%)
Brainstem	4 (21.1)	18 (31.0)
Basal ganglia	3 (15.8)	17 (29.3)
Thalamus	2 (10.5)	14 (24.1)
Spinal cord	3 (15.8)	13 (22.4)
Cerebellum	4 (21.1)	10 (17.2)
Optic nerve	3 (15.8)	10 (17.2)
Leotomenings	3 (15.8)	9 (15.5)
Periventricular area	4 (21.1)	8 (13.8)
Corpus callosum	3 (15.8)	6 (10.3)
Brachium pontis	1 (5.3)	5 (8.6)
Treatment		
First-line therapy		
IVMP	19 (100.0)	28 (48.3)
IVIG	10 (52.6)	33 (56.9)
PLEX	2 (10.5)	5 (8.6)
Second-line therapy		
RTX	3 (15.8)	15 (25.9)
CTX	0 (0.0)	1 (1.7)
AZA	2 (10.5)	3 (5.2)

*IVMP, intravenous methyl-prednisolone pulse; IVIG, intravenous immunoglobulins; PLEX, plasma exchange; RTX, rituximab; CTX, cyclophosphamide; AZA, azathioprine*.

* *Three cases did not presented the cortical lesions were unilateral or bilateral*.

### Anti-NMDAR-IgG^+^/MOG-IgG^+^ vs. Anti-NMDAR-IgG^−^/MOG-IgG^+^

Three observational studies (Wang et al., Hou et al., and Kunchok et al.) were enrolled in this section (15, 18, 20). The age of onset did not differ between anti-NMDAR-IgG^+^/MOG-IgG^+^ and anti-NMDAR-IgG^−^/MOG-IgG^+^ (median [IQR], Wang et al., 27.5 [24, 28.75] vs. 16 [11, 24]; Hou et al., 6 [5, 7] vs. 5 [3.4, 6.2]; Kunchok et al., 18 [13, 30] vs. 27.4 [10.6, 50.7], all *p* > 0.05). The data were not pooled, as only the median (IQR) was presented in these studies. Other demographics, such as the proportion of females, relapse, and preceding infection, were almost the same between these two groups. With regard to clinical features, demyelinating manifestations including optic neuritis were more common in anti-NMDAR-IgG^−^/MOG-IgG^+^ (anti-NMDAR-IgG^+^/MOG-IgG^+^ vs. anti-NMDAR-IgG^−^/MOG-IgG^+^, OR, 0.27; 95%CI, 0.09–0.83); however, encephalitic manifestations, including mental behavior, involuntary movement, and sleep disorder, always occurred in anti-NMDAR-IgG^+^/MOG-IgG^+^ (OR, 68.38; 95%CI, 1.36–3,434.37, OR, 57.86; 95%CI, 2.53–1,325.11; OR, 195.00; 95%CI, 7.07–5,380.15). The leptomeninges were more often invaded in anti-NMDAR-IgG^+^/MOG-IgG^+^ (OR, 7.32; 95%CI, 1.81–29.58), while other MRI changes did not differ between the two groups. No differences in follow-up mRS scores were observed between the two groups (median [IQR], Wang et al., 1 [1, 1] vs. 1 [1, 1]; Hou et al., 0 [0, 1] vs. 1 [1, 1], all *p* > 0.05). All of these results are presented in Table 2.

**Table 2:** Pooled analysis of the comparison between anti-NMDAR-IgG^+^/MOG-IgG^+^ and anti-NMDAR-IgG^−^/MOG-IgG^+^ or anti-NMDAR-IgG^+^/MOG-IgG^−^.

**Characteristics** **OR, 95%, CI**	**Involved studies**	**Anti-NMDAR-IgG** ^**+**^ **/MOG-IgG** ^**+**^ **vs. anti-NMDAR-IgG** ^**−**^ **/MOG-IgG** ^**+**^	**Involved studies**	**Anti-NMDAR-IgG** ^**+**^ **/MOG-IgG** ^**+**^ **vs. anti-NMDAR-IgG** ^**+**^ **/MOG-IgG** ^**−**^
Demographic				
Female	(15, 18, 20)	0.90, 0.18–4.45^#^	(18)	2.92, 0.48–17.86
Relapsed	(15, 18)	0.52, 0.02–14.85^#^	(16, 18)	**5.63, 1.75–18.09**
Preceding infection	(18)	0.28, 0.04–1.76	(16, 18)	**2.69, 1.03–7.02**
Clinical manifestations				
Encephalitic manifestations				
Seizures	(15, 18, 20)	2.52, 0.95–6.66	(16, 18)	0.68, 0.25–1.84
Mental behavior	(15, 18)	**68.38, 1.36–3,434.37** ^#^	(18)	1.53, 0.07–35.53
Sleep disorder	(18)	**195.00, 7.07–5,380.15**	(18)	0.78, 0.07–8.93
Sphincter dysfunction	(18)	0.27, 0.01–5.74	(18)	NA
Involuntary movement	(18)	**57.86, 2.53–1,325.11**	(18)	0.32, 0.05–1.90
Demyelinating manifestations				
Speech disorder	(15, 18)	2.10, 0.02–240.28^#^	(18)	1.43, 0.14–14.70
Optic neuritis	(15, 18, 20)	**0.27, 0.09**–**0.83**	(18)	NA
Paralysis	(15, 18)	1.40, 0.35–5.56	(18)	3.62, 0.64–20.41
Ataxia	(15, 18)	0.57, 0.11–2.87	(18)	0.21, 0.01–4.20
MRI				
Leptomeninges	(18, 20)	**7.32, 1.81–29.58**	(18)	4.17, 0.23–76.60
Cortical	(15, 18)	2.18, 0.54–8.81	(18)	4.44, 0.77–25.65
Subcortical	(15, 18)	2.53, 0.54–11.83	(18)	**116.60, 4.89–2,782.09**
Deep brain white matter	(15, 18)	0.13, 0.02–1.18	(18)	1.13, 0.04–30.81
Periventricular	(15, 18)	0.48, 0.07–3.37	(18)	0.45, 0.02–9.69
Corpus callosum	(15, 18)	1.42, 0.16–12.52	(18)	NA
Thalamus	(15, 18)	3.08, 0.61–15.49	(18)	4.80, 0.54–42.63
Basal ganglia	(18)	1.93, 0.34–10.77	(18)	**68.14, 2.99–1,554.27**
Brainstem	(15, 18)	2.04, 0.49–8.44	(18)	**24.09, 1.01–574.81**
Cerebellum	(18)	0.57, 0.05–5.88	(18)	4.17, 0.23–76.60
Spinal cord	(15, 18, 20)	1.09, 0.38–3.08	(18)	**24.09, 1.01–574.81**

# *Random-effects model computed by DerSimonian-Laird method. NA, no available. Bold text indicates the significant statistical difference*.

### Anti-NMDAR-IgG^+^/MOG-IgG^+^ vs. Anti-NMDAR-IgG^+^/MOG-IgG^−^

Two articles (Hou et al. and Zhang et al.) were included in this analysis (16, 18). There was no difference of the onset age between anti-NMDAR-IgG^+^/MOG-IgG^+^ and anti-NMDAR-IgG^+^/MOG-IgG^−^ (median [IQR], Hou et al., 6 [5, 7] vs. 5 [3.4, 6.2]; Zhang et al., 6.31 ± 3.82 vs. 7.60 ± 3.92, all *p* > 0.05]. The proportions of relapse and preceding infection in anti-NMDAR-IgG^+^/MOG-IgG^+^ were higher than those in anti-NMDAR-IgG^+^/MOG-IgG^−^ (anti-NMDAR-IgG^+^/MOG-IgG^+^ vs. anti-NMDAR-IgG^+^/MOG-IgG^−^, OR, 5.63; 95%CI, 1.75–18.09; OR, 2.69; 95%CI, 1.03–7.02). No significant differences in clinical features were observed between the two groups. In terms of MRI changes, subcortical regions, basal ganglia, brainstem, and spinal cord were always invaded in anti-NMDAR-IgG^+^/MOG-IgG^+^, rather than anti-NMDAR-IgG^+^/MOG-IgG^−^ (OR, 116.60; 95%CI, 4.89–2,782.09, OR, 68.14; 95%CI, 2.99–1,554.27, OR, 24.09; 95%CI, 1.01–574.81; OR, 24.09; 95%CI, 1.01–574.81). Higher follow-up mRS scores could be seen in anti-NMDAR-IgG^+^/MOG-IgG^−^ in the study by Hou et al. (median [IQR], anti-NMDAR-IgG^+^/MOG-IgG^+^ vs. anti-NMDAR-IgG^+^/MOG-IgG^−^, 0 [0, 1] vs. 2 [1, 3], *P* < 0.05). All of these results are presented in Table 2.

## Discussion

The coexistence of dual-positive autoimmune antibodies has garnered the attention of researchers owing to a series of atypical symptoms or lesions that cannot be explained by one antibody. Coexisting anti-NMDAR-IgG and MOG-IgG have become a research hotspot, as these two types of antibodies are more commonly seen in clinical settings than other autoimmune antibodies (2). Titulaer et al. first reported that 6% of NMDARE patients coexisted with serum MOG-IgG^+^ (7). However, with the growing number of similar studies published, we realized that the rate of these coexisting incidences may be underestimated, and the clinical features are not well-described. In doing so, we performed this secondary analysis to further investigate the incidence rates and clinical features of patients with coexisting anti-NMDAR-IgG^+^ and MOG-IgG^+^ to provide an overview of this disease.

The incidence rate of CSF anti-NMDAR-IgG^+^ in the serum MOG-IgG^+^ populations varied from 5 to 22.2% in previous studies (8, 15, 17, 19, 20). After pooling these results together, we found that approximately 9% of the serum MOG-IgG^+^ patients had coexisting CSF anti-NMDAR-IgG^+^ and 5% coexisted with NMDARE. Similarly, in patients with NMDARE, previous studies reported that the incidence rate of serum MOG-IgG^+^ ranged from 2.5 to 16.9 (5, 7, 16). The pooled analysis showed an incidence rate of 7%. Taken together, dual positivity of anti-NMDAR and MOG antibodies was very rare in the clinical setting, with an incidence of no more than 10% in patients with serum MOG-IgG^+^ or NMDARE.

Most of the patients with anti-NMDAR-IgG^+^/MOG-IgG^+^ (77.6%) presented with encephalitic manifestations during the first episode, in whom seizures accounted for 51.1%. Only 27.6% of patients presented with demyelinating manifestations at the first attack, of whom optic neuritis accounted for 68.8%. In addition, when comparing the clinical features of anti-NMDAR-IgG^−^/MOG-IgG^+^ and anti-NMDAR-IgG^+^/MOG-IgG^+^, anti-NMDAR-IgG^+^/MOG-IgG^+^ was associated with encephalitis (mental behavior, involuntary movement, and sleep disorder), and anti-NMDAR-IgG^−^/MOG-IgG^+^ was characterized by demyelinating manifestations (optic neuritis). However, when comparing anti-NMDAR-IgG^+^/MOG-IgG^+^ with anti-NMDAR-IgG^+^/MOG-IgG^−^, no difference in clinical features was observed. It is well-acknowledged that the most common presentation of MOG-AD is optic neuritis, occurring in 54–61% of patients (37–40). Meanwhile, ~90% of patients with NMDAREs have prominent psychiatric or behavioral symptoms (41). Hence, our results showed that the clinical presentations of the patients with anti-NMDAR-IgG^+^/MOG-IgG^+^ seemed to be more similar to NMDARE, rather than MOG-AD, indicating that MOG-IgG may only play an auxiliary role in anti-NMDAR-IgG^+^/MOG-IgG^+^.

Brain MRI changes revealed that leptomeninges were more often invaded in anti-NMDAR-IgG^+^/MOG-IgG^+^ than in anti-NMDAR-IgG^−^/MOG-IgG^+^, while the subcortical regions, basal ganglia, brainstem, and spinal cord were more likely to be destroyed in anti-NMDAR-IgG^+^/MOG-IgG^+^ compared with anti-NMDAR-IgG^+^/MOG-IgG^−^. The lesions in NMDARE are frequently found in the cortex, leptomeninges, and frontal, temporal, and limbic lobe lesions (2). MOG-AD has bilateral lesions predominantly in the brainstem, and over half of MOG-AD patients have T2 hyperintense lesions in the spinal cord (37). Our results demonstrated that anti-NMDAR-IgG^+^/MOG-IgG^+^ incorporated NMDARE as well as MOG-AD-associated brain MRI changes. The clinical subtypes of MOG-AD, UCCEE, and BFCCE have received considerable attention (42–45). In this study, 10/32 anti-NMDAR-IgG^+^/MOG-IgG^+^ patients presented with UCCEE, which was higher than the incidence calculated in MOG-IgG^+^ populations reported by Ogawa et al. (3/24) (44). These results suggest that the anti-NMDAR antibody seemed to contribute to the cortex destruction and epilepsy in MOG-IgG^+^ patients, which is also a characteristic of NMDARE. Meanwhile, 4/32 anti-NMDAR-IgG^+^/MOG-IgG^+^ patients displayed BFCCE in this study. Fujimori et al. (14) suggested that NMDARE might develop concurrently with anti-MOG antibody-associated BFCCE. However, the incidence of BFCCE in MOG-IgG^+^ patients is still lacking, and we cannot give a convincing conclusion regarding the association of BFCCE with anti-NMDAR antibody.

The relapse rate in the patients we collated was 63.4%, and the relapsed symptoms comprised encephalitic manifestations and half-half demyelinating manifestations. Notably, relapse invariably occurred in patients with anti-NMDAR-IgG^+^/MOG-IgG^+^, with a much higher rate in comparison to anti-NMDAR-IgG^+^/MOG-IgG^−^. A relapsing course was reported in 44–83% of patients with serum MOG-IgG^+^ and 7.5–7.0% of patients with CSF anti-NMDAR-IgG^+^ (1, 37, 38, 40, 46, 47). Thus, serum MOG-IgG^+^ may promote relapse in the presence of anti-NMDAR IgG^+^. Even so, patients with anti-NMDAR-IgG^+^/MOG-IgG^+^ were more likely to have a favorable functional outcome at follow-up. In addition, preceding infection was more commonly seen in anti-NMDAR-IgG^+^/MOG-IgG^+^ compared with anti-NMDAR-IgG^+^/MOG-IgG^−^, indicating that infection may be implicated in the dual positivity of antibodies.

This meta-analysis had several limitations. First and foremost, our pooled analysis only involved observational studies and case reports, and the inevitable bias in these types of studies might have impacted our overall conclusions. In addition, the sample size was too small to draw comprehensive conclusions. Finally, the comparison between groups relied only on the several studies included in the meta-analysis. These cohorts cannot be representative of the full NMDARE and MOG-AD spectra.

## Conclusions

Dual-positivity for anti-NMDAR and anti-MOG antibodies is not commonly encountered in clinical settings. In patients with this disease phenotype, the incidence rate of relapse was very high, but the functional outcome might not be poor. Although overlapping symptoms and imaging changes of NMDARE and MOG-AD were observed in these patients, the overall symptoms seemed to be more similar to NMDARE, rather than MOG-AD. In light of the limitations of this study, further larger epidemiologic studies and secondary analyses are needed to draw more convincing conclusions.

## Data Availability Statement

The original contributions presented in the study are included in the article/supplementary material, further inquiries can be directed to the corresponding author/s.

## Author Contributions

JD, XL, and ZT formulated the conception and design of the study, drafted the manuscript, and prepared the figures. XL and ZT completed the screening of the publications and made critical revisions of the manuscript. JD was responsible for the statistical analysis and wrote the manuscript. All authors contributed to the article and approved the submitted version.

## Conflict of Interest

The authors declare that the research was conducted in the absence of any commercial or financial relationships that could be construed as a potential conflict of interest.

## Publisher's Note

All claims expressed in this article are solely those of the authors and do not necessarily represent those of their affiliated organizations, or those of the publisher, the editors and the reviewers. Any product that may be evaluated in this article, or claim that may be made by its manufacturer, is not guaranteed or endorsed by the publisher.
